# Statistical modelling of an outcome variable with integrated multi-omics

**DOI:** 10.1186/s12859-025-06349-0

**Published:** 2025-12-24

**Authors:** He Li, Zander Gu, Said el Bouhaddani, Jeanine Houwing-Duistermaat

**Affiliations:** 1 Department of Mathematics, Radboud University, Heyendaalseweg, 6525 AJ Nijmegen, Gelderland The Netherlands; 2 Department of Mathematics and Computer Science, Eindhoven University of Technology, De Groene Loper, North Brabant 5612 AE Eindhoven, The Netherlands; 3 Medical Research Council Biostatistics Unit, University of Cambridge, Robinson Way, Cambridge, Cambridgeshire, CB2 0SR UK; 4 Julius Centre, UMC Utrecht, Universiteitsweg, 3584 CG Utrecht, Utrecht The Netherlands; 5 Population Health Research, King Abdullah International Medical Research Center, Mecca 22384 Jeddah, Saudi Arabia; 6 King Saud bin Abdulaziz University for Health Sciences, Mecca 22384 Jeddah, Saudi Arabia; 7 Department of Statistics, University of Leeds, Woodhouse Lane, Leeds, West Yorkshire, LS2 9JT UK

**Keywords:** Multivariate analysis, Low-dimensional representation, Latent variables, Polygenic score, Metabolomics

## Abstract

**Background:**

In studies that aim to model the relationship between an outcome variable and multiple omics datasets, it is often desirable to reduce the dimensionality of these datasets or to represent one omics dataset in terms of another. Several approaches exist for this purpose, including univariate methods such as polygenic scores, and multivariate methods. Multivariate approaches offer advantages by producing lower-dimensional integrative scores, capturing joint structures across datasets, and filtering out dataset-specific noise. In this paper, we describe one univariate and two multivariate methods, and evaluate their performance through simulations involving two correlated multivariate normally distributed omics datasets, as well as a combination of one multivariate normal and one fixed categorical dataset.

**Results:**

We assess method performance using the root mean squared error (RMSE) when modelling the outcome variable as a function of the reduced omics representations. Multivariate methods generally perform well, particularly when a slightly higher number of components is used for integration. They outperform the univariate method in scenarios involving two normally distributed omics datasets and perform comparably in settings with one normal and one categorical dataset. In real data applications, including two metabolomics datasets from TwinsUK and a metabolomics-genetic dataset from ORCADES, all methods show similar performance in modelling body mass index.

**Conclusions:**

Multivariate methods provide a valuable framework for summarizing multi-omics datasets into low-dimensional components suitable for outcome modelling. Even in the presence of non-normal data, these methods offer a promising alternative to high-dimensional univariate approaches.

**Supplementary Information:**

The online version contains supplementary material available at 10.1186/s12859-025-06349-0.

## Background

Multi-omics datasets, which include data from genetic markers, transcriptomics, proteomics, and metabolomics, are increasingly available in epidemiological studies. Statistical analysis of multiple datasets jointly has the potential to provide insights in underlying biological mechanisms. However, the high-dimensionality and complex correlation structures of these datasets pose significant challenges for statistical analysis [1]. To extract meaningful insights, summarizing multi-omics data into lower-dimensional representations that retain relevant biological information is needed. In this paper, the specific goal is to represent one omics dataset by another in a low-dimensional form. In the second step, the low-dimensional representation is included in a model for an outcome variable. For instance, we are interested in the effect of the genetic parts of metabolites on the outcome variable, necessitating the summarization of a metabolomics dataset in terms of genetic markers. While both univariate and multivariate methods exist to achieve this, selecting the most appropriate approach for a specific scenario remains an open question.

A univariate method to describe one omics dataset by another involves performing linear regression for each feature of the first omics dataset. For a single outcome variable, this approach is commonly used in genetics. Specifically, the polygenic score (PGS), also known as the polygenic risk score (PRS), is a linear combination of a subject’s genotype and the estimated effects of multiple genetic variants on an individual’s phenotype [2]. These estimates are constructed using the summary statistics from large genome-wide association studies. Similarly, a linear combination for each feature of the first omics dataset can be computed based on the features of the second. Another example of a univariate is a transcriptome-wide association study (TWAS) where the association between traits and gene expressions is modelled. One approach for such an analysis is PrediXcan [3, 4]. Alternative univariate approaches include regularized regression methods, such as ridge regression and least absolute shrinkage and selection operator (LASSO). Ridge regression addresses highly correlated variables by shrinking non-influential coefficients towards small values [5]. In contrast, LASSO [6] shrinks some coefficients to zero, effectively selecting variables. This shrinkage reduces dimensionality and may improve interpretation, making LASSO a preferable method for obtaining an omics representation of another omics dataset.

Univariate approaches result in a representation with a dimension equal to that of the dependent omics dataset. To further reduce the dimension, multivariate approaches can be considered. These methods integrate two datasets and reduce dimensionality simultaneously by identifying associations and patterns among variables within and between the two omics datasets [7]. Hence, a compressed representation of the one omic in terms of the other can be obtained. Several multivariate approaches have been developed. For example, the partial least squares (PLS) model [8], its sparse extension [9], and its probabilistic extension [10] are used to jointly model the two omics datasets from the same samples. These methods decompose the variation in each dataset into a joint part and a residual part and do not model data specific variation [11]. Since the two omics datasets might have different characteristics, quantification of this data-specific variation can be beneficial. Two-way orthogonal partial least squares (O2PLS) [11, 12] and its probabilistic version PO2PLS [1] were developed to include this data-specific variation. Further, while sequential approaches are fast, they may lead to overfitting [1] in small datasets. In such cases, PO2PLS, which estimates the parameters simultaneously by maximizing the likelihood function, might be preferable. From these multivariate models, the joint components of one omics dataset in terms of the second can be used as a low-dimensional representation.

Other types of methods that can analyse multiple omics datasets are machine and deep learning approaches [13]. These integration methods also capture non-linear relationships. Typically, these methods concatenate multiple datasets into a single large matrix while keeping the same set of samples, meaning that in contrast to O2PLS and PO2PLS, the heterogeneity between datasets is not modelled. Other non-linear methods transform each omics dataset into a simpler representation, such as kernel learning [14]. Hence, these methods analyse the datasets separately rather than jointly.

The obtained low dimension representation referred to as “integrate-scores" in the rest of the paper, will be included in a model for the outcome variable. Thus we consider two-stage approaches, in the first stage, the integrate-scores are derived using a univariate or multivariate method and in the second stage the outcome is modelled with the integrate-scores as covariates. For the univariate method, we propose a LASSO approach to represent each feature of an omics dataset based on the other dataset. For the multivariate methods, we use O2PLS and PO2PLS to decompose the two omics datasets into joint, data-specific and residual parts, with the joint component of one dataset serving as the integrate-scores. In the second stage, we use either linear or LASSO regression with the integrate-scores as independent variables, depending on the number of the integrate-scores.

Our methods will be illustrated by datasets from the TwinsUK study [15] and the Orkney Complex Disease Study (ORCADES) [16]. In TwinsUK, we use one metabolomics dataset to represent another, from which we obtain the integrate-scores. In ORCADES, we consider genetic SNPs and metabolomics datasets. In both studies, the outcome of interest is the body mass index (BMI). To model BMI based on a summarised representation of the two omics datasets, we apply the two-stage approach: we first reduce the dimensionality of the metabolomics or genetic datasets, and then use the resulting summaries to model BMI. To compare the performance of the methods, we design simulation studies based on three settings: (i) two normally distributed omics datasets, (ii) one normally distributed dataset and a continuous multidimensional dataset (eigengenes), and (iii) one normally distributed dataset and a categorical dataset (genotypes). Our simulation considers various scenarios, including different sample sizes, dimensions, and noise levels. The performance of the methods will be evaluated by RMSE and $R^2$ of the outcome variable.

The contributions of this paper are as follows. Firstly, we propose our two-stage approaches for modelling an outcome variable using the low-dimensional integrate-scores derived from two omics datasets. Secondly, we conduct an extensive simulation study to compare the performance of the univariate and multivariate methods used in the first stage. Thirdly, we provide guidance on method selection for different data scenarios, including varying levels of noise, dimensionality and sample size. Fourthly, we demonstrate the practical utility of the proposed approaches by analysing omics data from two population-based studies. The R code including generating and analysing data is available on GitHub (https://github.com/heli-math/2-StageApproaches_Multi-Omics ).

This paper is organised as follows. Section 2 introduces the two-stage approach, the univariate and multivariate methods, the simulation designs, the datasets and the pre-processing procedures. Section 3 shows the outcomes of simulation and data analysis. At last, the discussions and conclusions are in Sects. 4 and 5.

## Methods

Let *x* and *y* be two random vectors of length *P* and *Q* ($Q<P$), respectively. Multiple samples are collected in the variables *X* ($N \times P$) and *Y* ($N \times Q$), representing two omics datasets, where *N* denotes the number of subjects and *P* and *Q* denote the number of variables in *X* and *Y*, respectively. The features in *X* may be correlated with those in *Y*. A third random variable *Z* ($N \times 1$) represents the outcome. We will model the relationship between *Z* and the part of *Y* that can be represented by *X*, referred to as the “integrate-scores". For example, if *X* represents genetic markers and *Y* represents metabolomics data, we might be interested in the relationship between *Z* and the genetic representation of the omics datasets.

We consider two-stage methods to model the outcome variable *Z*. At the first stage, we focus on obtaining the integrate-scores, which will serve as the explanatory variables in the second stage. To derive these integrate-scores, we consider three approaches, namely one univariate and two multivariate methods. Note that our aim is to predict the *Y* variables from *X*, rather than to perform causal inference. In the second stage, we model the outcome using linear regression to estimate the effects of the integrate-scores on the outcome variable. Depending on the dimensionality (the number of predictors versus samples), we apply the regularised regression techniques, such as LASSO regression, to avoid overfitting.

### Statistical methods

#### Stage 1: Constructing integrate-scores for *Y* based on *X*

The univariate method fits a separate regression for each variable in *Y*,  producing integrate-scores with the same dimension as *Y*,  namely *Q*. As for the multivariate methods, we consider O2PLS and PO2PLS to model *X* and *Y* jointly. Here, O2PLS is an algorithmic approach and PO2PLS is a likelihood-based approach that makes the assumption of multivariate normal distributed the variables *x* and *y*.

*Univariate approach: Omic-PGS* Here, we model each random variable in *Y* as a linear combination of *X*. To deal with the potentially high dimensionality of *X*, LASSO regression is used to shrink some coefficients towards zero. For *N* subjects, omic feature *q*, each regression coefficient vector $\beta _q$ ($q=1,\cdots ,Q$, with length *P*) can be estimated by

1 $$\begin{aligned} {\hat{\beta }}_{q}^{LASSO} = \underset{\beta _q}{\arg \min } \sum _{i=1}^N ( Y_{iq} - X_{i} \beta _{q} )^2+\lambda \sum _{p=1}^P | \beta _{pq} |, \end{aligned}$$

where $\beta _{pq}$ $(p=1, \cdots , P)$ represents the association of the *p*th feature in *X* on the *q*th feature in *Y*. The degree of shrinkage is controlled by the penalty parameter $\lambda$, typically chosen through cross-validation. The resulting integrate-scores are given by ${\hat{Y}} = X {\hat{\beta }}^{LASSO}$, where ${\hat{\beta }}^{LASSO} = ({\hat{\beta }}^{LASSO}_1,{\hat{\beta }}^{LASSO}_2, \cdots , {\hat{\beta }}^{LASSO}_Q)$.

*Multivariate approaches* Univariate approaches model the correlation between *X* and *Y*, while ignoring the correlations within each dataset and the variances of the features in *X*. Since multivariate approaches model the whole covariance structure, they might better summarise the data. The model underlying the two considered multivariate methods O2PLS and PO2PLS is as follows.

2 $$\begin{aligned} \begin{aligned}&X = TW^\top + T_\perp W_\perp ^\top + E, \\&Y = UC^\top + U_\perp C_\perp ^\top + F, \\&U=TB+H, \end{aligned} \end{aligned}$$

where $W \, (P \times r)$ and $C \, (Q \times r)$ are the loading matrices for the joint spaces of *X* and *Y*, with *r* usually a small number. $W_\perp \, (P \times r_x)$ and $C_\perp \, (Q \times r_y)$ are the loading matrices for the specific parts. The loading values indicate the relative importance of each genetic or omic variable in forming the corresponding components. The $r \times r$ diagonal matrix *B* models the relationship between the joint components *T* and *U*. The estimate of *T* represents a linear combination of the variables in *X*. We name the *r*-dimensional ${\hat{T}}$ integrate-scores obtained by multivariate methods.

Since O2PLS is an algorithm approach, it is not necessary to specify the distribution of the latent variables. On the other hand, PO2PLS is a probabilistic approach and assumes that all latent variables follow the normal distribution. Since the joint likelihood of the data and latent variables factorizes in different parts, the likelihood can be maximized by using the EM algorithm. Let *e* and *f* be the residuals of the two submodels for *x* and *y*. We assume that *e* and *f* are independently normally distributed with zero mean and covariance matrices $\sigma _e^2 I_p$ and $\sigma _f^2 I_q$, respectively. Note that *E* and *F* comprise the *N* latent copies of *e* and *f*, respectively. The latent variables $t, t_\perp , u_\perp , h$ follow multivariate normal distributions with zero means and diagonal covariance matrices $\Sigma _t, \Sigma _{t_\perp }, \Sigma _{u_\perp }$ and $\Sigma _h$, respectively. The covariance matrix of the joint variable *u* is given by $\Sigma _u = B^\top \Sigma _t B + \Sigma _h$. All parameters are collected in $\theta := \left\{ W, W_{\perp }, C, C_{\perp }, B, \Sigma _t, \Sigma _{t_{\perp }}, \Sigma _{u_{\perp }}, \Sigma _h, \sigma _e^2, \sigma _f^2 \right\}$, parametrising the joint distribution $(x,y) \sim {\mathcal {N}} (0, \Sigma _{\theta })$. For details on the estimation procedure see Bouhaddani et al. [1].

To obtain the estimates by O2PLS and PO2PLS model, we use the R package “OmicsPLS” [17] and “PO2PLS” on GitHub (https://github.com/selbouhaddani/PO2PLS), respectively. Both methods can choose optimal component numbers via cross-validation [18]. In addition to the optimal value for *r*, we also propose multivariate models with a larger number of components $r^+$ than *r*. For example between *r* and $\min (P-r_x, Q-r_y)$, which is the maximum possible value.

#### Stage 2: Modelling the outcome based on the integrate-scores

From the obtained integrate-scores ${\hat{Y}}$ (with dimension $N \times Q$ in univariate approach) or ${\hat{T}}$ (with dimension $N \times r$ in multivariate approaches) in the first stage, the second stage aims to find their relationship with the outcome *Z*.

For cases with a small number of integrate-scores (i.e., small *Q* or *r*), we model the outcome *Z* in terms of integrate-scores using linear prediction: ${\hat{Z}} = {\hat{Y}} {\hat{\gamma }}$ or ${\hat{Z}} = {\hat{T}} {\hat{\gamma }}$. Here, the estimate ${\hat{\gamma }}$ describes the effect of the integrate-scores on the outcome variable. For a large number of integrate-scores, we apply LASSO regression to obtain the regression parameter estimate ${\hat{\gamma }}^{LASSO}$. Apart from regularisation, LASSO can also identify variables with non-zero coefficients.

Since modelling outcome *Z* requires two stages, we use separate training and test datasets: a first-stage training dataset of size *N* to generate integrate-scores, a second-stage training dataset of size $N_h$ to obtain ${\hat{Y}}$ and train the second stage, and another test dataset of size $N_k$ to evaluate performance in stage 2.

### Simulation study

#### Simulation designs

We consider three simulation designs to compare the performance of the univariate and multivariate methods. Each design is described in detail below, including whether datasets *X* is treated as random or fixed, along with its parameter settings.

For Design I, we generate data according to the PO2PLS model, where the row variables *x* and *y* are random and repeatedly generated. This approach enables us to evaluate the performance of the methods when data varies across repeated experiments. For example, metabolomics can be treated as random because metabolite levels vary over time, and they might fluctuate due to factors such as environmental and physiological conditions, as well as measurement and technical variability.

Besides Design I, where the datasets are generated as multivariate normal random variables, we also consider cases where the covariate *X* is fixed. In Design II, the dataset *X* is generated once using the PO2PLS software. In Design III, *X* is a genotype generated by the simulator Cosi2 [19].

The performance of the three methods will be evaluated by root mean squared error (RMSE) and coefficient of determination ($R^2$) of *Z*. RMSE is calculated in both training and test datasets to assess model fit and detect potential overfitting, while $R^2=1-SSE/SST$ is calculated on the test data to quantify the proportion of variance in *Z* explained by the predictions.

*Design I: PO2PLS* The sample pairs *x* ($1 \times P$) and *y* ($1 \times Q$) are jointly generated under various scenarios from the PO2PLS model. Specifically, the heterogeneity in this design is quantified by the variance proportion of *h* over *u*, i.e., the ratio of the joint residual variance $\Sigma _h$ to the total joint variance $\Sigma _u$. Given the component counts *r*, $r_x$ and $r_y$, the elements in the diagonal matrix *B* are $r, r-1, \cdots , 1$.

In this design, the variance of *x* and *y* is composed of joint, specific and random parts. Given the variance of the joint and specific parts, we set the proportions of variance in *e* and *f* as $\alpha _e$ and $\alpha _f$ relative to the variance of *x* and *y*, respectively. The proportion of variance in *h* is set as $\alpha _{tu}$ of that in *t*, reflecting the impact of heterogeneity in the joint components. In the process of generating *x* and *y*, we record the joint components *t*, then the outcome variable *z* is subsequently generated by

$$\begin{aligned} z = a_0 + t a^\top +g, \end{aligned}$$

with $a_0$ the intercept, *a* row-vector of size *r*, and the elements of *g* follow a normal distribution with zero mean. The outcome variable *z* is linearly determined by the joint component *t*, with $\alpha _g$ representing the proportion of variance in *z* explained by *t*. The variables *x* and *y* are repeatedly generated to comprise the datasets *X* and *Y* of size *N*.

*Design II: PCA* Now we consider *X* ($N \times P$) fixed, and then *Y* ($N \times Q$) is generated based on the principal components of the fixed *X*.

In this context, *X* is generated once from the PO2PLS model under the same conditions as previously described. After fixing matrix *X*, the other dataset *Y* is then generated through the components specified as $T = XW$. The weight matrix *W* ($P \times r$) is composed of the top *r* PCA loadings of *X*. Consequently, matrix *T* ($N \times r$) contains *r* components, which are linear combinations of the columns of *X*. The matrix *C* ($Q \times r$) is categorial, allowing some groups of *X* (e.g., a pathway) to influence *Y*, each column represents one of the *r* groups. The residual *F* represents unexplained variation in *Y*, while $TC^\top$ captures the structured relationship between *X* and *Y*. Here, *F* is repeatedly generated while $TC^\top$ is fixed. The outcome variable *Z* is then generated by a linear representation from *Y*. The model for *Y* and *Z* is given by

3 $$\begin{aligned} \begin{aligned} Y&= TC^\top +F, \\ Z&= TC^\top l + G. \end{aligned} \end{aligned}$$

The elements of the random variable *G* are independently and identically distributed. The vector łwith length *Q* is defined as the first principal loading vector of the matrix $TC^\top$, mapping the components of *Y* to the outcome variable *Z*.

*Design III: Cosi2* Design III is similar to II, but now *X* includes categorical variables representing genotypes. The dataset *X* is generated once using the Cosi2 simulator and is fixed. We simulate $2 {\widetilde{N}}=2\left( N+N_h+N_k\right)$ haplotypes for a length of $1.25\text {e}^5$ regions in base pairs. We can identify the mutation positions among the total columns by setting other features such as a mutation rate as $1.5\text {e}^{-8}$ per base pair per generation, and a recombination rate of $1.0\text {e}^{-8}$.

Since humans are diploid, we randomly pair the simulated haplotypes. Furthermore, we retain only the variants from the total region with an allele frequency of at least 1%. Ultimately, we obtain the matrix *X*, which consists of 2234 columns comprising the values 0, 1 and 2. Then datasets *Y* and *Z* are generated in the same way as Design II.

#### Parameter settings

For the three designs proposed above, various scenarios are considered. We explore different sample sizes (*N*=200, 2000) and variable counts (*P*=100, 2000; *Q*=25, 30). Note that we mainly vary the dimension *P* of the *X* dataset, since *Y* is represented by *X* through the integrate-scores. Consequently, changes in *P* directly affects the model complexity, while increasing *Q* might increase the prediction performance because more covariates are included in the model for *Z*. Since the methods are two-stage, the training (*N* samples) and test datasets ($N_h, N_k=1000$ samples) are generated simultaneously from the same design and are used separately in the estimating and prediction processes.

In Design I, we consider *x* and *y* with low and high noise levels ($\alpha _e$=10%, 95%; $\alpha _f$=10%, 60%) and heterogeneity levels ($\alpha _{tu}=10\%, 40\%$). The outcome variable *z* maintains a fixed noise level ($\alpha _g$ = 10%). The component counts $r, r_x, r_y$ are all set to 5.

In Design II and III, considering *X* is fixed, we calculate the sample variance in matrix *X* and then set the proportions $\alpha _F$ and $\alpha _G$ as the noise levels to obtain *Y* and *Z*.

### Data application

We applied the univariate and multivariate approaches to TwinsUK and ORCADES studies. In TwinsUK study, two metabolomics datasets were modelled representing the case of two normally distributed datasets. In ORCADES, a genetic data set, namely SNPs, and a metabolomics dataset will be modelled. We will calculate eigengenes which correspond to the case of one normally distributed random dataset and a continuous covariate. Finally, we will model the metabolomics and the genetic markers corresponding to the case of one normally distributed random dataset and a categorical covariate. Note that since *X* is a genetic dataset, our methods will provide a genetic part of the metabolites. For both studies, in the second stage, we will model BMI as function of the obtained integrate-scores.

#### The twinsUK cohort

**Fig. 1:**
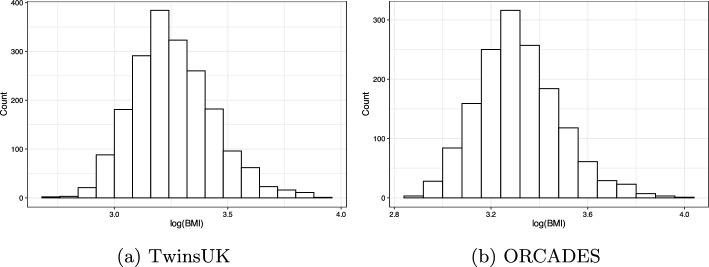
For TwinsUK and ORCADES: histograms of log(BMI). Age and sex effects are removed by linear regression

From the TwinsUK cohort, a longitudinal study, we integrate the matched samples from the two metabolomics datasets and select the visit for which most metabolomics measurements are available. Since there are only a few male participants, we only take the female participants. This results in a total of 1944 samples. The log-transformed BMI values adjusted for age for these participants are given in Fig. 1. Concerning the omics datasets, we have access to 901 metabolites in Metabolon and 239 metabolites in Brainshake. In Metabolon, 145 metabolites were observed in less than 80% of the samples and thus were excluded from further analysis [20]. All samples were measured more than 70% of their metabolites in both Metabolon and Brainshake. In the end, we have a dataset comprising 1944 female individuals with measurements for 756 Metabolon metabolites and 213 Brainshake metabolites. These variables were processed by Box-Cox transformation. Further, since age might have effects on the metabolites [21], we applied linear regression on each metabolite in terms of age and took the residuals as age-corrected metabolite measurements for modelling.

#### The ORCADES cohort

The ORCADES cohort consists of 1885 inhabitants from Orkney. As a population isolated from the remote Orkney Isles in northern Scotland, the cohort exhibits a distinctive genetic background resulting from its long-standing geographical isolation [22, 23]. And family structure is present in the cohort. To remove the family structure in the data, we estimated the kinship coefficient between individuals from the genomic data using KING-robust [24] and excluded one individual from each pair with a kinship coefficient greater than 0.25 (i.e., the first-degree relatives such as parents, full siblings and children are removed). The individual excluded from a pair was determined in such a way that the number of participants was maximized (using PLINK 2 [25]). After kinship-based pruning, our study comprised 1490 individuals. All these 1490 samples had missing genotype rates $< 0.1$. The log-transformed BMI values of the participants adjusted for age, sex and relatedness are given in Fig. 1.

We filtered out SNPs that had minor allele frequency (MAF) below 0.05. The 5314423 SNPs after the MAF filter were used to construct PGSs for both omic features and the outcome. For the integrative methods, we further pruned the SNPs with a $r^2$ threshold of 0.5 and removed SNPs that were not close to a gene (within 50k base pair). This yields 261644 SNPs. The remaining 261644 SNPs were used for the SNP-based analyses (and referred to as the SNP data). For the gene-based analyses, we aggregated the SNPs around the same gene using PCA [1], and the first few genomic principal components (GPCs) that explained at least 80% of the variance in each gene were taken. This resulted in a dataset with 95185 GPCs (referred to as the GPC data). The GPCs were approximately normally distributed.

The metabolomic data were measured using the high throughput NMR metabolomics assay (Nightingale Health Ltd., Helsinki, Finland), consisting of 225 metabolite measures in molar concentration units. In this paper, we restrict our analysis to the 108 metabolites which overlap with the ones in the GWAS study of metabolomics conducted in [26]. We first excluded metabolites with a missing rate greater than $5 \%$ (0 metabolites excluded), and then replaced the zeros in the data with half of the lowest observed level for this metabolite, following the quality control steps in [27]. Then we performed univariate regression of BMI on each metabolite in ORCADES and selected the 87 metabolites that had a nominal *p*-value of less than 0.05 for further analysis. A Box-Cox transformation [28] with parameter 1/4 was then performed to reduce skewness [12]. Metabolite measures were set to missing based on a z-score cut-off of 6 [29]. The missing data were imputed once by chained equations (mice) [30]. Lastly, the imputed dataset was corrected for age and sex using multiple regression and the residuals were used.

To further reduce the dimensions of the genetic data, we took the top 1000 SNPs with the smallest *p*-values for each metabolite based on the GWAS summary statistics in [26]. This resulted in a union of 11272 SNPs (1148 GPCs).

## Results

### Simulation results

We apply the univariate, O2PLS and PO2PLS methods to the data generated under the designs described in the previous Sect. 3.2. For each scenario 500 replicates were generated. For the two multivariate methods O2PLS and PO2PLS, in addition to the number of component *r* used to generate the data, we also consider a larger number namely $r^+ = 12$. This number is between *r* and the maximum components $Q-\text {max}(r_x, r_y)$. In the plots, these two methods are denoted as “method-*r*” and “method-$r^+$”. Here, RMSE is preferred, since it reflects both bias and variance compared with $R^2$. Box-plots of the RMSE of the parameter estimators are shown. A reference line which represents the true noise in the outcome variable is given as well. Note that the reference lines might differ across scenarios due to variations in the parameters under different conditions. We also made box plots of the coefficient of determination $R^2$ which are shown in the supplement. Since methods appear to perform similarly for the two heterogeneity levels, we only show the results for $\alpha _{tu}=10\%$. The results of $\alpha _{tu}=40\%$ can be found in the supplement.

**Fig. 2:**
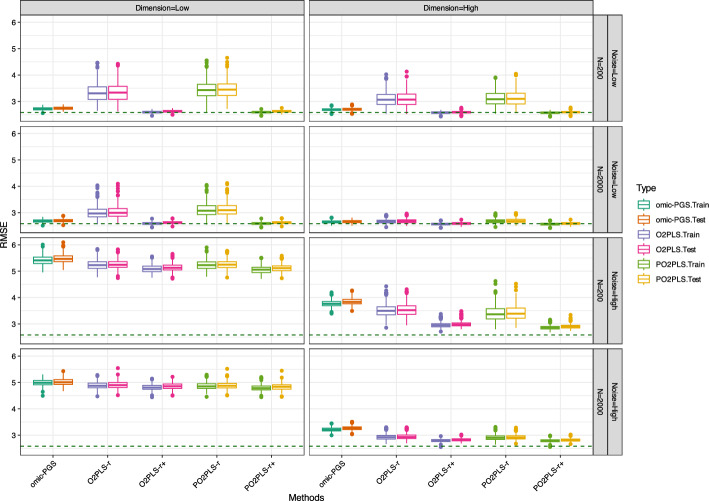
Simulation results for Design I: RMSE of training and test datasets stratified by method and scenario. The reference line at 2.58 represents the median RMSE when using the true parameter values. “method-*r*” and “method-$r^+$” represent small and large components of multivariate methods, respectively

The performance of the five methods for Design I are shown in Fig. 2. In general, the multivariate methods with $r^+$ components perform best for this design. For the low noise setting (top layer), all methods perform similarly for high dimension and large sample size. Specifically, when using fewer components *r*, the median RMSE values on the test sets are comparable across methods: 2.66 for omic-PGS, 2.67 for O2PLS, and 2.68 for PO2PLS. When using more components $r^+$, RMSE of both O2PLS and PO2PLS slightly decrease to 2.59. While for low dimension and low noise, the two multivariate methods with *r* components perform worse than the multivariate methods with $r^+$ components and the univariate method. For example, when using small *N*, the median RMSE for O2PLS and PO2PLS decreases from 3.33 and 3.45 to 2.60 and 2.61. The other three methods (omic-PGS, O2PLS and PO2PLS using $r^+$ components) show similar performance for these settings. For high noise (bottom layer), all methods perform similarly for the low dimension and large sample size settings. For the low dimension and small sample size and the high dimension and large sample size, the univariate performs a little worse than the multivariate methods. Specifically, in the low-dimensional, high noise, small *N* scenario, the median RMSE values for omic-PGS, O2PLS, and PO2PLS are 5.47, 5.24, and 5.25, respectively. When using more components $r^+$, the RMSE values for O2PLS and PO2PLS further decrease to 5.12 and 5.11. For the high-dimensional and small sample size setting, the multivariate with $r+$ components perform better than the other three methods. The median RMSE values for omic-PGS, O2PLS, and PO2PLS are 3.84, 3.52 and 3.39. When more components $r^+$ are used, the RMSE values for O2PLS and PO2PLS decrease significantly to 2.98 and 2.89.

**Fig. 3:**
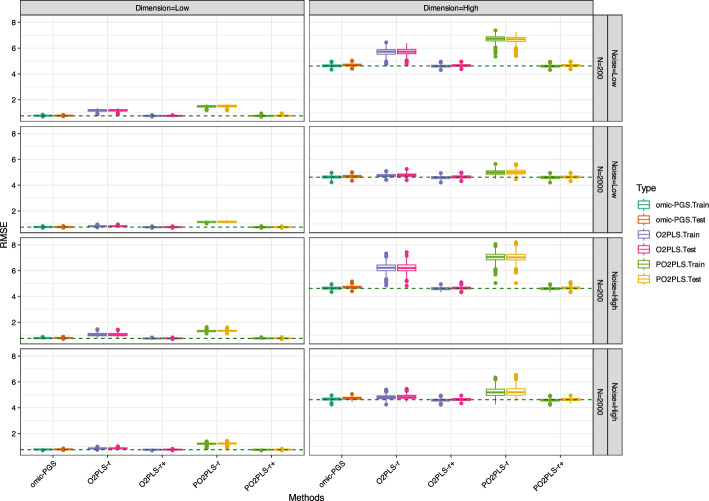
Simulation results for Design II: RMSE of training and test datasets stratified by method and scenario. The reference lines at 0.75 (low dimension) and 4.62 (high dimension) indicate the median RMSE when using the true parameter values. “method-*r*” and “method-$r^+$” represent small and large components of multivariate methods, respectively

The performance of the five methods for Design II are shown in Fig. 3. In general, the multivariate methods with $r^+$ components and the univariate methods perform best for this scenario. For the low noise setting (top layer), all methods perform similarly for all settings except for low dimension and a small sample size, where the two multivariate methods with *r* components perform worse than the other three methods. The medians of the multivariate methods decrease from 1.19 and 1.52 to 0.76 and 0.76. For the high noise setting (bottom layer), all methods perform similarly across most scenarios, except in the low-dimensional, small sample case. In this setting, the multivariate methods with *r* components perform worse than the other three methods. For instance, under high noise with high-dimensional and small *N*, the RMSE for the multivariate methods with *r* components are 6.20 and 7.30. In contrast, the RMSE values for omic-PGS, O2PLS, and PO2PLS with $r^+$ components are 4.72, 4.69 and 4.69, respectively.

**Fig. 4:**
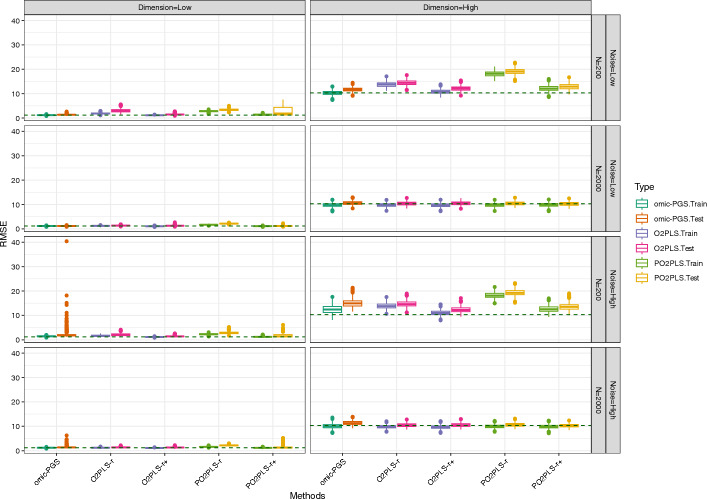
Simulation results for Design III: RMSE of training and test datasets stratified by method and scenario. The reference lines at 1.19 (low dimension) and 10.33 (high dimension) represent the median RMSE when using the true parameter values. “method-*r*” and “method-$r^+$” represent small and large components of multivariate methods, respectively

The performance of the five methods for Design III are shown in Fig. 4. In general, the multivariate methods with $r^+$ components and the univariate methods perform best for this scenario. For the low noise setting (top layer), all methods perform similarly for all settings except for low dimension and small sample size, where the multivariate PO2PLS method with *r* components performs worse than the other four methods. In this high-dimensional, low noise, small *N* scenario, the RMSE of the multivariate methods using *r* are 14.43 and 12.12, which are higher than those of omic-PGS, O2PLS and PO2PLS using $r^+$ components, at 11.70, 12.86, and 12.12, respectively. In the high noise setting (bottom layer), all methods show similar median error levels across most scenarios, except for the high-dimensional and small sample size setting. For the latter setting, the multivariate methods with $r^+$ components perform better than the other three. Specifically, the RMSE of O2PLS and PO2PLS with $r^+$ components are 12.41 and 12.64, lower than the other three 14.96, 14.65 and 19.24. Note that for low dimension and small *N*, although the median RMSE for the univariate is similar to the median RMSE of the other methods, the variance is much larger.

Note that in practice, *r* is not known and will be determined by cross-validation or scree plots. However, as the simulation results suggest, more components result in better performance. Indeed, we verified this for Design I (see the supplementary materials).

### Application to twinsUK and ORCADES datasets

#### Procedures of data application

We applied univariate and integrative methods to the metabolomics datasets available in the TwinsUK and to the metabolomics and SNP data in the ORCADES cohort. For TwinsUK, Brainshake is the independent omics dataset and Metabolon dependent. Concerning the SNP data, we will consider normally distributed eigengenes calculated from the SNPs and the categorical SNPs as independent omics data. The performance of the methods is assessed by the RMSE.

We randomly split the TwinsUK cohort into discovery (1000 samples) and target (1014 samples) sets, and the ORCADES cohort into discovery (1000 samples) and target (490 samples) sets.

Since the numbers of samples are relatively small, we use the discovery datasets to train both the first and second stages and derive the integrate-scores. The target datasets are then used to model BMI.

For TwinsUK cohort, the joint and specific components were set to 6, 4 and 0 by scree plots. For GPCs in ORCADES cohort, the number of joint, genomic-specific, and omic-specific components were set to 6, 5 and 0. For SNPs of ORCADES cohort, the components were 8, 2 and 1. We will also consider models with a larger number of joint components, namely 12 components.

#### Results of data application

For TwinsUK datasets, the performances of the models for BMI with as covariates Brainshake features (*Y*) represented by Metabolon features (*X*) are shown in Table 1.

**Table 1:** TwinsUK: RMSE for predicting log(BMI) using Brainshake integrate-scores based on Metabolon

	${\hat{r}}$		${\hat{r}}^+$	
	Discovery	Target	Discovery	Target
Univariate	0.1251	0.1472	0.1251	0.1472
O2PLS	0.1665	0.1718	0.1663	0.1675
PO2PLS	0.1691	0.1778	0.1648	0.1746

*Note:*The numbers of components${\hat{r}}$are determined by the scree plots, and${\hat{r}}^+$is given between${\hat{r}}$and the maximum number of components

For this study, the univariate method performs a little better than the multivariate methods. When using more components, the multivariate methods are slightly improved. When using the original Brainshake metabolites in a LASSO model for BMI, the RMSE value appears to be similar, namely 0.1637 in the target set. In Table S3 of the supplementary materials, we report the top ten Brainshake metabolites contributing to the BMI obtained by univariate, the two multivariate methods with ${\hat{r}}^+$, and by using the original metabolites. Here, for the univariate method, the metabolites are ranked using the estimated coefficients ${\hat{\gamma }}^{LASSO}$, whereas for the multivariate methods, they are ranked by the product $C {\hat{B}}^{-1} {\hat{\gamma }}^{LASSO}$. When comparing the top ten metabolites represented by Metabolon with the top ten metabolites obtained using the original metabolites, it appears that “L-LDL-C” is the only metabolite shared among all methods (see Table S3 in the supplementary materials). In addition, the univariate method share more metabolites with the top ten based on the original metabolites.

For ORCADES, the performances of the methods in explaining BMI using the SNPs and GPCs representatives of metabolomics are shown in Table 2. The top ten metabolites for the various approaches are given in Table S4 in the supplementary materials. Compared with using the results based on the original metabolites, the overlapping metabolites are “DHA” and “Val” for univariate and “FAw3”, “MUFA” for O2PLS. The top ten metabolites obtained by PO2PLS do not overlap with the original top ten list. Since the metabolites are represented by genetic markers, the selected metabolites might be more heritable. To investigate this, we used the heritability estimates given in Supplementary Dataset3 by [27]. From the original metabolites, the heritability of five metabolites is given in the dataset. These metabolites have a mean heritability of 0.37. From the top ten based on the univariate, the heritability of three metabolites are given by [27]. They have a mean heritability of 0.40. From the top ten based on O2PLS, there is one metabolite with a heritability of 0.46. None of the metabolites of the top ten obtained with PO2PLS was in the table of the paper [27].

**Table 2:** ORCADES: RMSE for predicting log(BMI) using GPCs integrate-scores based on pre-screened SNPs

	${\hat{r}}$		${\hat{r}}^+$	
	Discovery	Target	Discovery	Target
omic-PGS (SNPs)	0.1683	0.1741	0.1683	0.1741
O2PLS (SNPs)	0.1675	0.1766	0.1626	0.1760
PO2PLS (SNPs)	0.1700	0.1742	0.1704	0.1737
omic-PGS (GPCs)	0.1627	0.1769	0.1627	0.1769
O2PLS (GPCs)	0.1681	0.1781	0.1641	0.1777
PO2PLS (GPCs)	0.1700	0.1745	0.1704	0.1738

## Discussion

We proposed various methods to describe one omics dataset in another one by a low-dimensional representation. These low-dimensional representations are subsequently incorporated into a model for an outcome variable. Via a simulation study, we evaluated the performance of the considered methods for constructing the integrate-scores in their ability to explain the outcome. Specifically, we compared a univariate method which models each omic feature individually and two multivariate methods across various scenarios. For the multivariate methods, we determined the optimal number of components by cross-validation. In addition, we considered a larger number of components, namely a value in between the optimal and the maximum possible number. In general, using more components improved the performance of the multivariate methods and for most situations, the multivariate with additional components and the univariate perform similarly. Hence, multivariate methods might be preferred since they are more parsimonious.

The univariate and multivariate methods were applied to omics data from two studies, namely TwinsUK and ORCADES. In TwinsUK, we have access to two metabolomics datasets, and in ORCADES we have a genetics and metabolomics dataset. For both studies, BMI is used as the outcome variable. In both studies, all methods perform similarly. For TwinsUK, for the univariate method there are 213 scores to be included in the model and LASSO has to be used, while O2PLS and PO2PLS summarize the data in only 6 (*r*) or 12 ($r^+$) scores. For ORCADES, for the univariate method omic-PGS there are 87 scores to be included in the model and LASSO has to be used, while O2PLS and PO2PLS summarize the data in only 8 and 6 (*r*) for SNPs and eigengenes or 12 ($r^+$) for SNPs and eigengenes scores. For this application, our approaches estimate the genetic part of the metabolites. Indeed, the heritability of the top ten metabolites from the univariate and from O2PLS appears to be higher, namely 0.40 (univariate) and 0.46 (O2PLS) versus 0.37 (original metabolites).

The univariate method applies LASSO regression to model one omic dataset by the other and obtain the integrate-scores. The multivariate methods perform well for many considered scenarios. However, they assume normal distributions. O2PLS is less sensitive for deviations than PO2PLS. However, when we have SNPs with small frequencies, this method might perform worse. On the other hand in the simulation, we noticed that the univariate method does not perform well for low-dimensional data and high noise. An advantage of the multivariate methods is that they include a data-specific part, so the joint components can be better estimated. This results in better and or lower dimensional representations. A drawback is that PO2PLS might be computationally expensive. On the other hand, it performs better in small datasets [1].

Based on the simulation results and data application, we provide the following guidelines for selecting the best method to construct the integrate-scores, considering prediction accuracy, computational time and the number of parameters to be estimated. (i) For multivariate normally distributed data, PO2PLS or O2PLS should be used with a larger number of components than the optimal one based on cross-validation. The advantage of the latter is that the computational time is lower. On the other hand, for small sample sizes, PO2PLS is more accurate. (ii) For a categorical dataset *X*, we also recommend using O2PLS or PO2PLS with a larger number of components than the optimum based on cross-validation. For many settings, the univariate omic-PGS performs similarly as the multivariate. However, it does not perform well in low dimensions with high noise. Moreover, typically more parameters need to be estimated in the model for the outcome.

In our simulation and data applications, it appears that using $r^+$ components is beneficial. However, selecting the best number of components in terms of explaining the variance of the outcome remains a question. To circumvent this issue, joint modelling of the two omics-dataset and the outcome might be used [31]. However, this might result in overfitting of the model for the outcome and is also computationally challenging. Moreover, it will be necessary to fit a new model for each outcome variable.

Our methods are applicable in many situations. For example, in the case of genetic data, one might be only interested in the heritable part of an omics dataset. Another application is when one dataset is more expensive or invasive to obtain than the other one, and the data are longitudinally measured. Our methodology requires measuring both datasets for just one time point, and for the other time points the scores can be used. For example, there appears to be a strong correlation between cerebrospinal fluid (CSF) and serum metabolites [32], suggesting that serum metabolomics can partly reflect CSF metabolic states. As CSF collection is invasive and costly, serum data provide a practical alternative. Also, when the numbers of observations are small, including two correlated high-dimensional omics datasets using regularisation on the parameters of the omics features might not give stable results. Moreover, in such cases, the correlation between the two datasets is not well modelled, and this may hamper interpretation.

In this paper, we made some choices that might be further investigated. For example, LASSO is applied as an univariate method because it only has one tuning parameter. Other methods such as elastic net that including both $L_1$ and $L_2$ penalties might be good alternatives. Indeed, for gene expression, recent works suggest that elastic net better captures the underlying genetic architecture [33]. Further, when analysing the ORCADES data, we selected 1000 SNPs per metabolite. The number of 1000 SNPs is arbitrary. A data-driven selection process might improve the results. However, the optimal selection procedure might vary across the univariate and multivariate methods.

We also restricted ourselves to linear relationships among the outcome variable and the omics datasets, while some machine and deep learning methods are available. Such methods may better capture the complexity of biological systems [34]. However, most of these methods do not address the heterogeneity present when using multiple omics datasets, since they just stack the datasets. Recently, a new approach based on autoencoders has been proposed, which considers both shared and dataset-specific variation [35].

There are several potential directions for future research that build on our current work. Firstly, choosing appropriate component counts to balance capturing sufficient signal variance and avoiding model mis-specification is a topic for future research. Secondly, future work could also use longitudinal models to study the components over time. An insight into longitudinal principal components modelling can be found in [36], though this work focuses on single datasets rather than multi-omics. Thirdly, our work focuses on modelling one outcome variable through the integration of two omics datasets. The proposed framework can be straightforwardly applied to multiple low-dimensional outcomes, as the second stage models the linear relationship between the outcomes and the integrate-scores. The method can also be applied when there are multiple omics datasets. However, the role of the two omics datasets in this paper is not the same. Two *Y* datasets are straightforward since they require performing step one twice, i.e., for each *Y* dataset. Two *X* datasets might need a new model and estimation method since the model needs to be adapted here. For example, we may be interested in the joint part of the two *X* datasets or in the joint and data specific parts.

## Conclusions

To obtain a low dimensional representation of an omics dataset in terms of another omics dataset, we showed the multivariate methods perform well. Advantages of multivariate methods are that they summarise the data in a smaller number of components and that they can filter out data-specific variation. Moreover in all the considered settings they are stable. This in contrast to the univariate method which appears to be unstable for low dimensions and noisy data. The good performance of the multivariate methods is also illustrated by the two data applications.

## Additional file

Supplementary file 1.

## Data Availability

The datasets analysed in this study are not publicly available due to participant privacy and data use agreements. Access to the TwinsUK data is available upon application through the TwinsUK Research Access Committee. Further information and application details can be found at: https://twinsuk.ac.uk/researchers/data-access-costs/. Access to the ORCADES data is available upon request via VIKING GENES. For more information, please visit: https://viking.ed.ac.uk.

## Abbreviations

BMI: Body mass index
CSF: Cerebrospinal fluid
GPCs: Genomic principal components
LASSO: Least absolute shrinkage and selection operator
MAF: Minor allele frequency
NMR: Nuclear magnetic resonance
O2PLS: Two-way orthogonal partial least square
ORCADES: Orkney complex disease study
PCA: Principal component analysis
PGS: Polygenic score
PLS: Partial least square
PO2PLS: Probabilistic two-way orthogonal partial least square
PRS: Polygenic risk score
RMSE: Root mean squared error
SNP: Single nucleotide polymorphism
SSE: Sum of squares error
SST: Sum of squares total
TWAS: Transcriptome-wide association study
