# Supplementary material for: Statistical modelling of an outcome variable with integrated multi-omics
Source: BMC Bioinformatics. 2025 Dec 24;27:26. doi: 10.1186/s12859-025-06349-0 (PMC12859906; doi:10.1186/s12859-025-06349-0)
Supplement: Supplementary file 1 [file 12859_2025_6349_MOESM1_ESM.pdf]

Supplementary for "Statistical Modelling of an Outcome Variable with Integrated Multi-Omics"

He Li, Zander Gu, Said el Bouhaddani, Jeanine Houwing-Duistermaat

Introduction

This document is the supplement for the main article "Statistical Modelling of an Outcome Variable with Integrated Multi-Omics". It is structured into four parts:

In Section S1, we provide a conceptual figure illustrating the structure of multivariate methods.

In Section S2, we provide an additional simulation for Design I. The counts of components are selected via cross-validation. The added simulation is denoted as "method-CV".

In Section S3, the simulation outcomes and real data analysis in R^2 are provided. Compared with RMSE, R^2 provides a scale-independent measure that reflects the proportion of variance in the outcome explained by the model, whereas RMSE reflects both bias and variance.

In Section S4, we present the outcomes under a high level of heterogeneity.

In Section S5, we explore the insights into the behaviour of the multivariate methods and to study their performance when more latent variation is captured.

In Section S6, we provide additional results from the data application: the top ten metabolites identified by different methods.

S1. Structure of multivariate methods

In this conceptual figure, the joint components T and U are correlated. The outcome variable Z is directly modelled on T in our proposed framework, and the correlation between Y and Z can be further modelled through T.

S2. Selected components via cross-validation under Design I

The jitter plot in Figure S2 illustrates how the components selected by cross-validation vary across different scenarios. The true numbers of components used to generate the data are $r = r_x = r_y = 5$. In the high-noise setting (the bottom layer), cross-validation tends to select fewer joint components (r) and more data-specific components (r_x and r_y, especially r_y). In the high dimensional setting, r_y is consistently selected to be larger.

The performance of the seven methods for Design I measured by RMSE are shown in Figure S3. The performance of multivariate methods using "method-r" and "method-r^+" have been discussed in the main text, here we focus on the "method-CV". The numbers of components for "method-CV" are dynamically determined by cross-validation, as illustrated in Figure S2. Overall, the performance of "method-CV" depends on the components

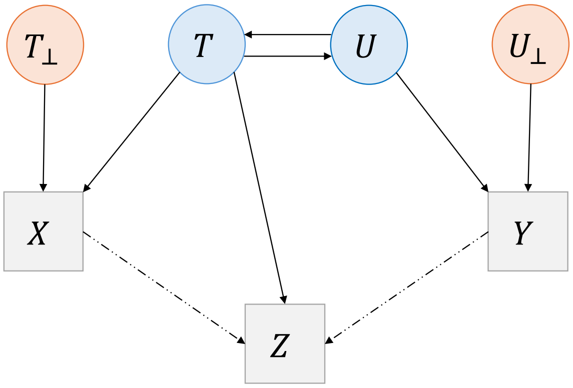

Figure S1: **Structure of multivariate methods on outcome modelling.** The squares are observable datasets, while the circles are latent variables.

selection. For example, in the low-noise setting (top layer), "method-CV" shows greater deviation compared with the other multivariate methods because the selected component numbers are dispersed rather than centred around a value. Under low dimensionality, low noise and small sample size (top-left block), "method-CV" performs slightly better than "method-r" but worse than "method-r^+", which is consistent with its selected component numbers lying between r and r^+ (see e S2). In other settings, however, it performs the worst, particularly under high dimensionality and high noise with larger sample sizes (bottom-right block), where the median RMSE for both O2PLS and PO2PLS reaches 3.44, significantly higher than those obtained using r and r^+.

S3. Simulation and Data Application Results by R^2

The simulation results measured by R^2 for Design I, II and III are shown in Figure S4, S5 and S6, respectively. In general, multivariate methods using more components r^+ perform as well as or better than omic-PGS. In contrast, multivariate methods with fewer components r tend to perform worse.

The results of data application expressed by R^2 are presented in Table S1 and S2. In the TwinsUK study (Table S1), multivariate methods using $\hat{r}^+$ have higher R^2 than those using $\hat{r}$. In ORCADES study (Table S2), the multivariate methods have negative R^2 values.

Table S1: TwinsUK: R^2 for test set in predicting log(BMI) using Brainshake integrate-scores based on Metabolon.

	$\hat{r}$	$\hat{r}^+$
omic-PGS	24.20	24.20
O2PLS	6.94	12.54
PO2PLS	1.68	4.81

Note: The numbers in the table are percentages of BMI explained (adjusted-$R^2 \times 100$). Negative adjusted-R^2s are recorded as 0.

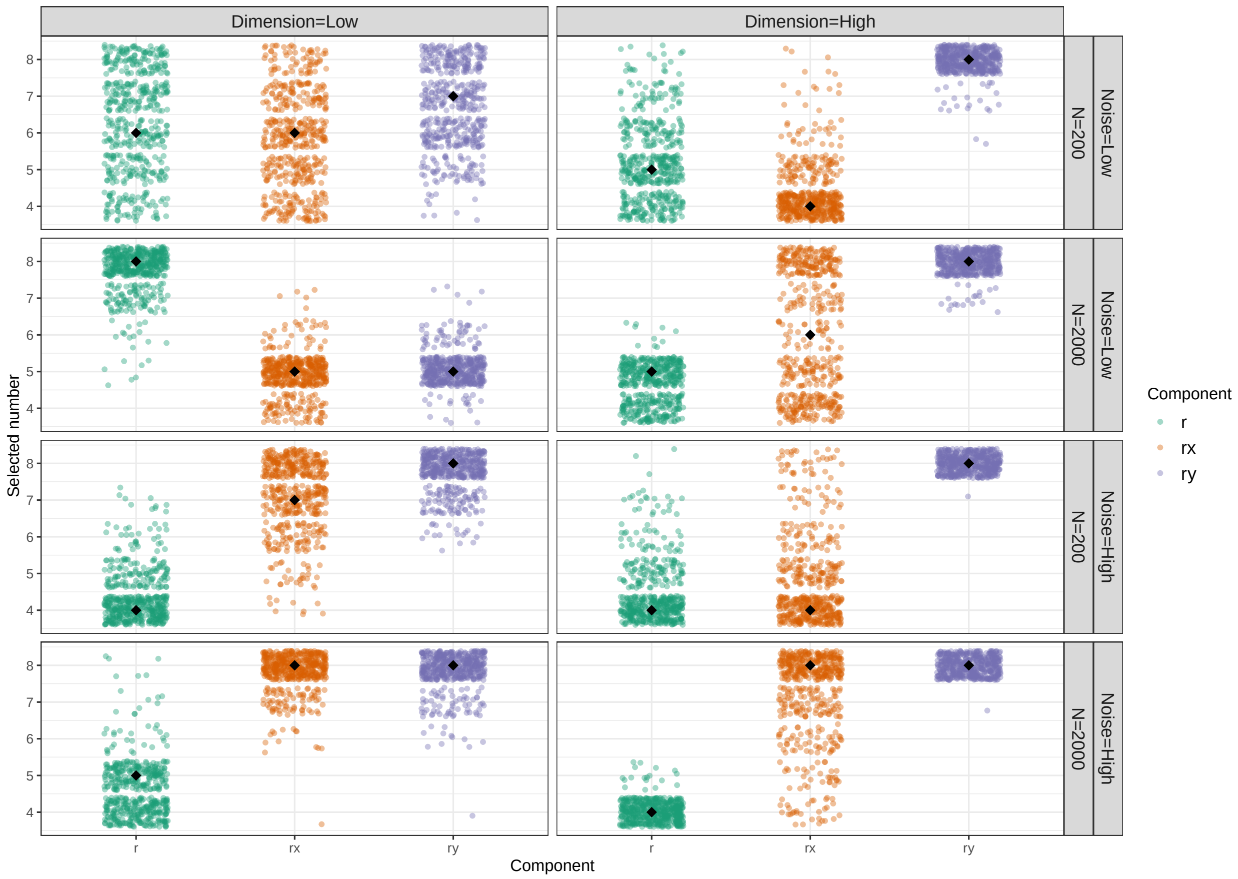

Figure S2: **Selected components by cross-validation under Design I.** The grid is defined as $r, r_x, r_y \in [4, 8]$. The black dots indicate the medians of the selected components across the corresponding scenarios.

S4. Simulation Results of High Heterogeneity Levels

Here, we set the heterogeneity level as $\alpha_{tu} = 40\%$, the outcomes are shown in Figure S7. The results indicates that the methods perform similarly under low or high heterogeneity levels.

S5. Simulation Results of Extra Large Components

For $r^{++} = 20$, the results are shown in Figure S8. In Design I, the three methods perform similarly under low noise (top layer). Under high noise, PO2PLS performs comparably to omic-PGS in the low-dimensional setting and outperforms it in the high-dimensional setting, while O2PLS perform worse than the other two methods. In Design III, the three methods again perform similarly in the low-dimensional setting. However, in the high-dimensional case, O2PLS performs worse, and PO2PLS shows a higher number of outliers.

S6. Data Analysis

Here, the detected ten Brainshake metabolites by different methods are provided in Table S3.

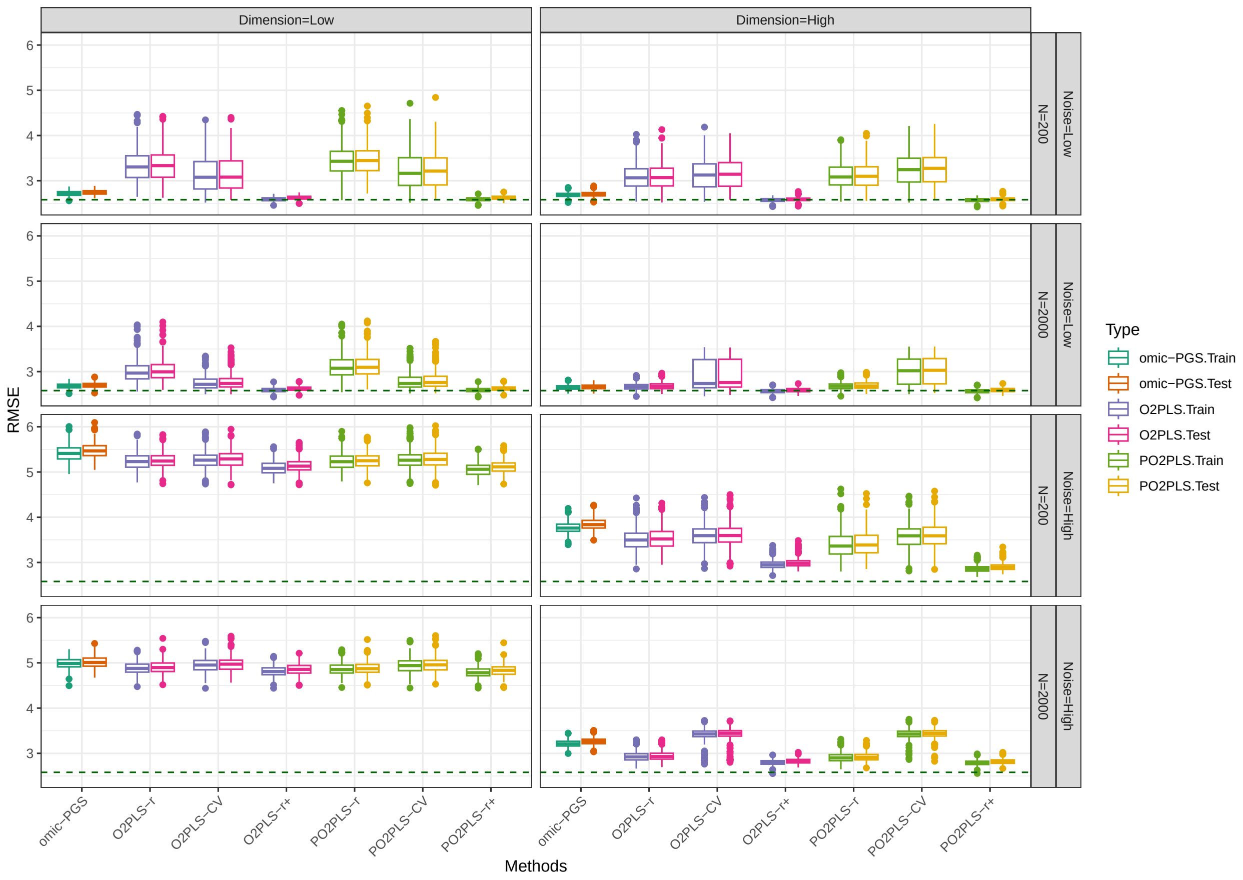

Figure S3: **Simulation results for Design I: RMSE of training and test datasets stratified by method and scenario.** The reference line at 2.58 represents the median RMSE when using the true parameter values. The x-axis labels "method-r", "method-CV" and "method-r^+" represent small, cross-validation-selected and large components of multivariate methods, respectively.

Table S2: ORCADES: R^2 for test set in predicting log(BMI) using SNPs and GPCs.

	$\hat{r}$	$\hat{r}^+$
omic-PGS (SNPs)	0.14	0.14
O2PLS (SNPs)	0	0
PO2PLS (SNPs)	0	0
omic-PGS (GPCs)	0	0
O2PLS (GPCs)	0	0
PO2PLS (GPCs)	0	0

Note: The numbers in the table are percentages of BMI explained (adjusted-$R^2 \times 100$). Negative adjusted-R^2s are recorded as 0.

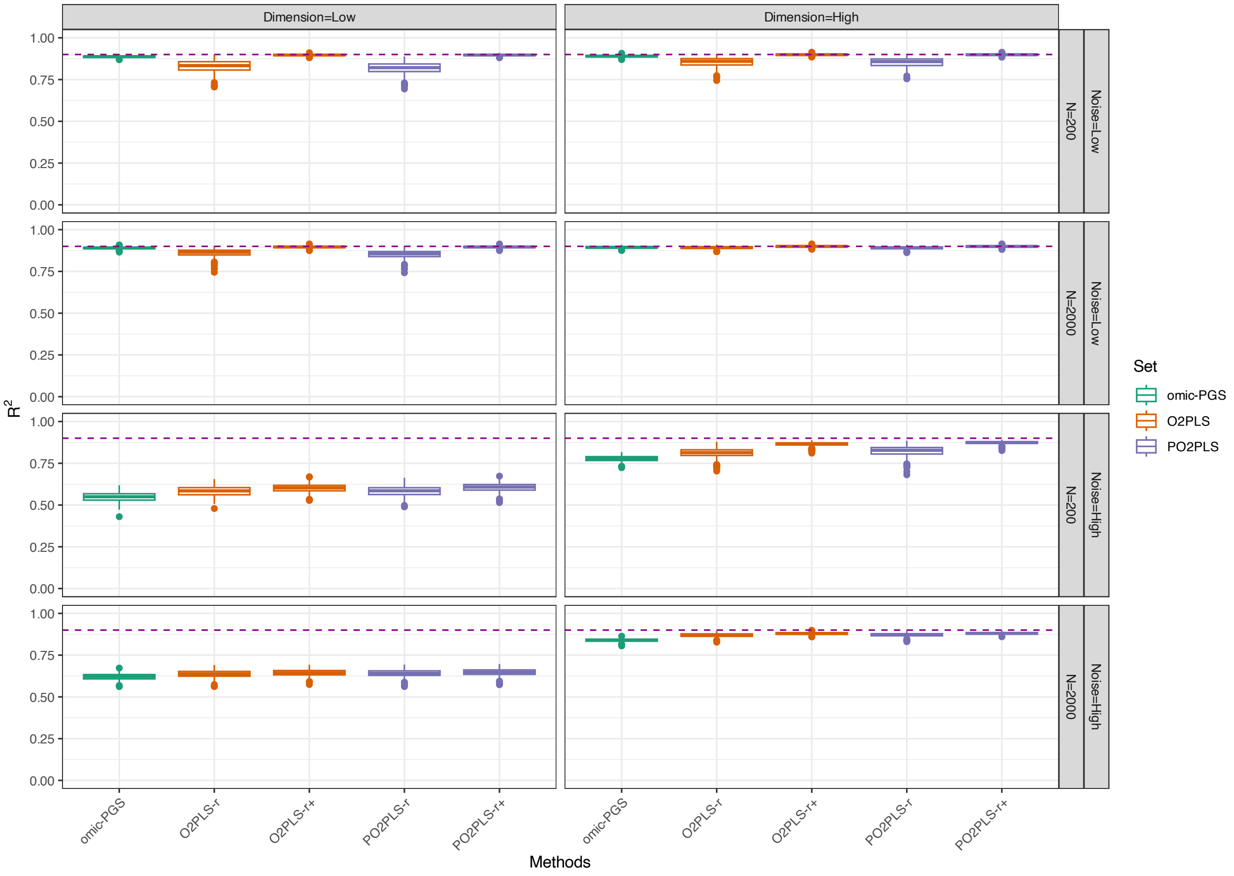

Figure S4: R^2 **of test datasets stratified by method and scenario in Design I.** The reference line at 0.9 represents the proportion of variance explained by the non-random component of the errors.

Table S3: TwinsUK: detected top ten Brainshake metabolites among different methods.

	Detected top ten metabolites
Original metabolites	**L-HDL-PL**, IDL-TG, **L-HDL-CE**, L-HDL-C, PUFA/FA, M-LDL-C, **XS-VLDL-C**, **L-LDL-C**, M-VLDL-C, S-VLDL-C
Univariate	**L-LDL-C**, **XS-VLDL-C**, **L-HDL-PL**, S-HDL-CE, SFA/FA, S-VLDL-TG, XL-HDL-C, L-HDL-CE, L-LDL-CE, S-LDL-C
O2PLS-$\hat{r}^+$	L-HDL-C, L-HDL-PL, **L-LDL-C**, SFA/FA, M-VLDL-CE, S-VLDL-TG, M-LDL-PL, FAw3/FA, **L-HDL-CE**, M-HDL-TG
PO2PLS-$\hat{r}^+$	**L-LDL-C**, SFA/FA, M-LDL-PL, M-LDL-TG, M-VLDL-CE, IDL-C, M-LDL-CE, S-HDL-CE, S-LDL-C, S-LDL-CE

Note: Original metabolites represent the actual measured values, rather than the estimated integrate-scores.

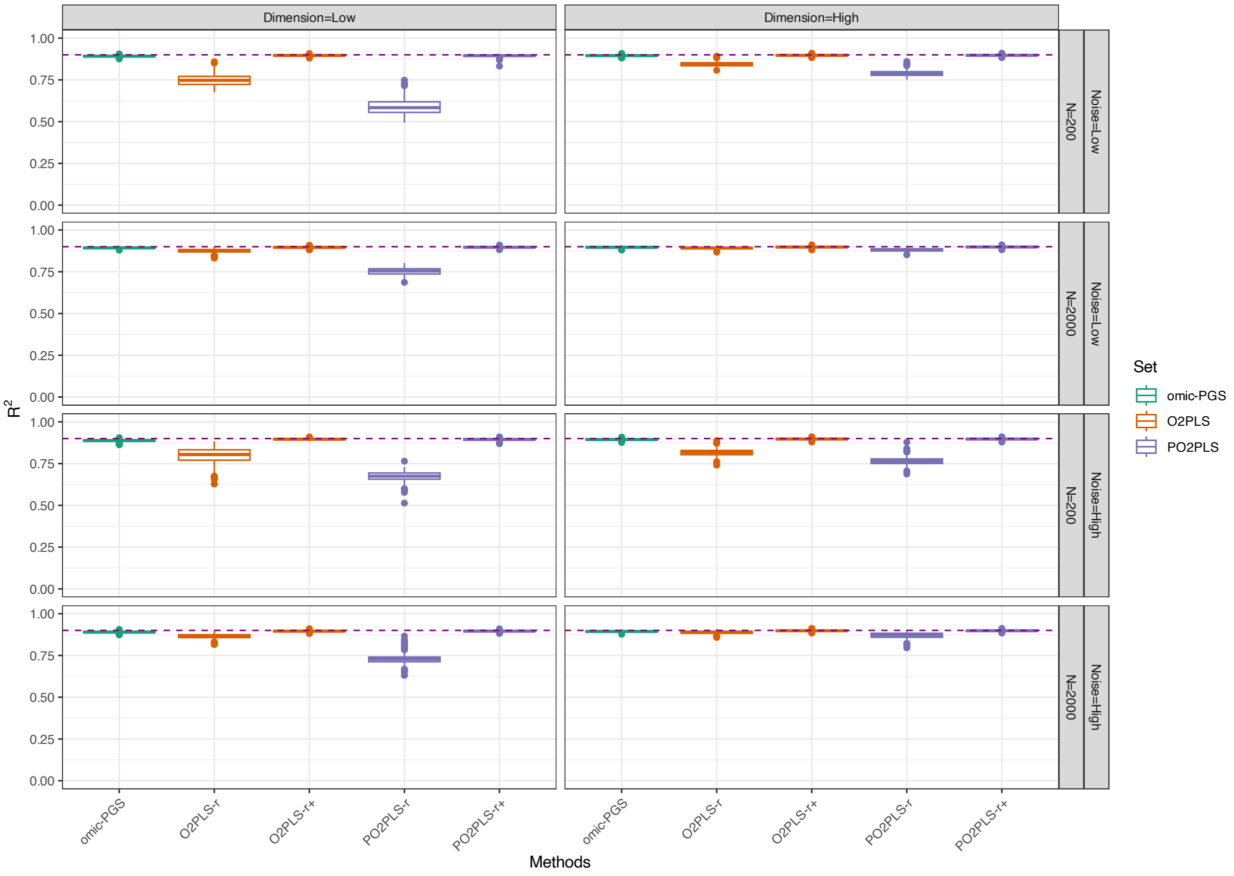

Figure S5: R^2 **of test datasets stratified by method and scenario in Design II.** The reference line at 0.9 represents the proportion of variance explained by the non-random component of the errors.

Table S4: ORCADES: detected top ten metabolites among different methods.

	Detected top ten metabolites
Original metabolites	Phe, Tyr, **DHA**, His, Alb, **Val**, Gp, **FAw3**, **MUFA**, XL.HDL.TG
omic-PGS	SM, Leu, **DHA**, Cit, M.LDL.PL, XS.VLDL.PL, **Val**, M.HDL.PL, IDL.FC, LDL.D
O2PLS-$\hat{r}^+$	L.HDL.PL, **MUFA**, S.VLDL.C, ApoA1, L.HDL.C, L.HDL.CE, IDL.FC, **FAw3**, M.VLDL.CE, S.VLDL.TG
PO2PLS-$\hat{r}^+$	SM, L.HDL.FC, ApoA1, M.VLDL.FC, M.VLDL.CE, L.VLDL.PL, L.VLDL.C, XL.HDL.P, L.VLDL.FC, XL.HDL.LE

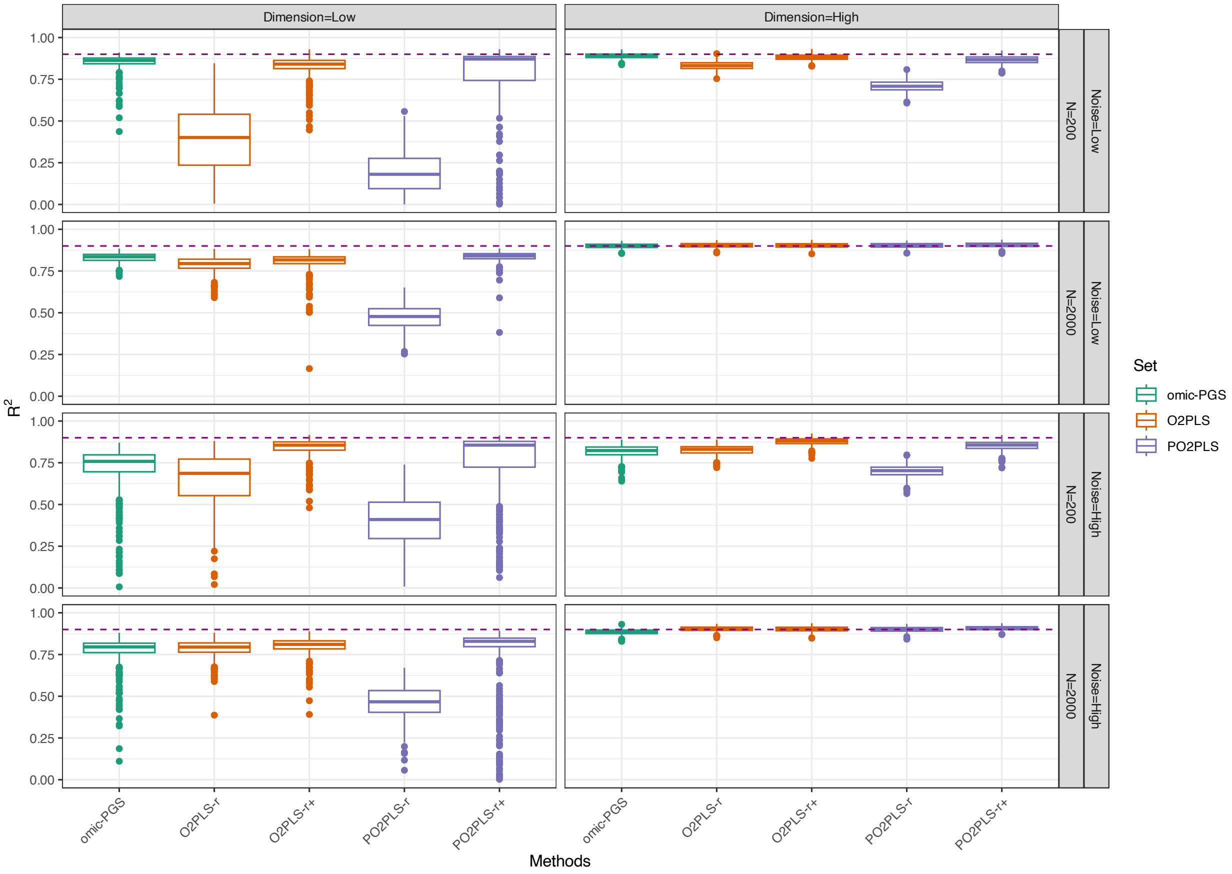

Figure S6: R^2 **of test datasets stratified by method and scenario in Design III.** The reference line at 0.9 represents the proportion of variance explained by the non-random component of the errors.

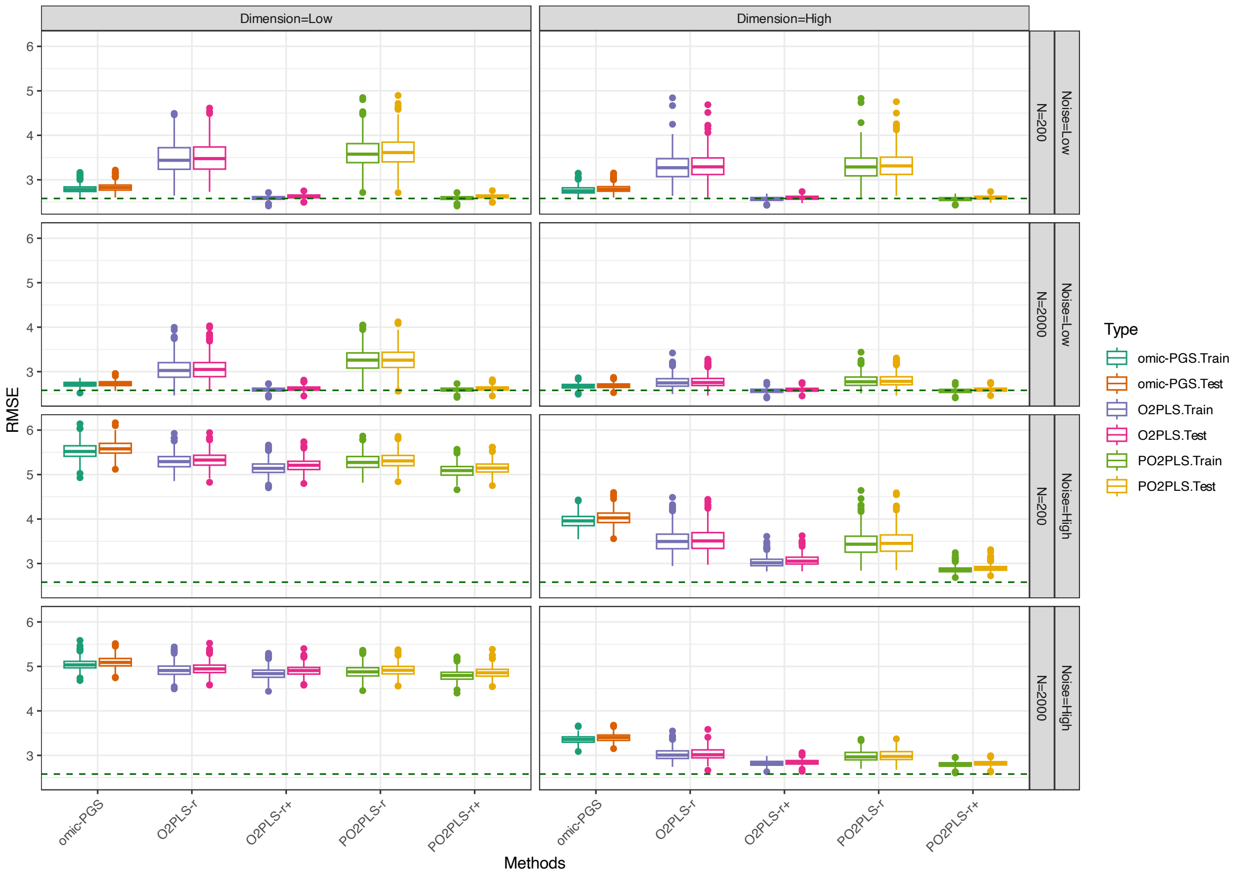

Figure S7: **RMSE of training and test datasets stratified by method and scenario in Design I**, under a high heterogeneity level $\alpha_{tu} = 40\%$. The reference line represents the true errors.

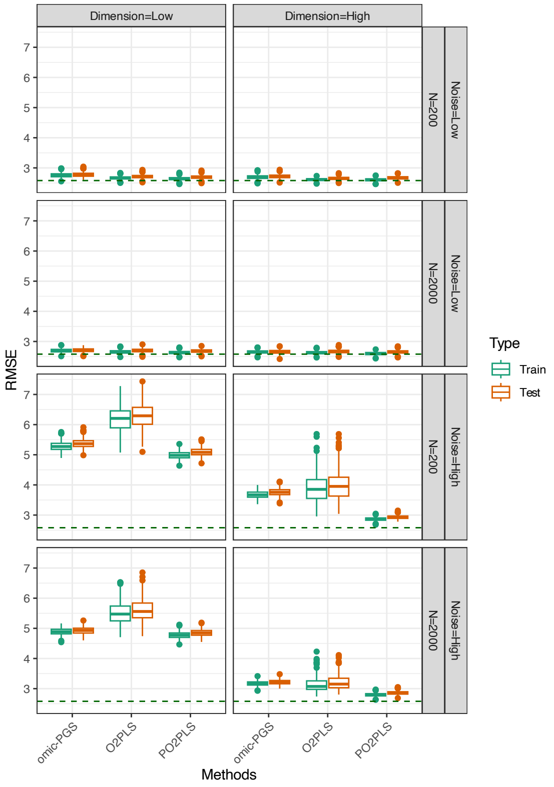

(a) Design I with $r^{++} = 20$

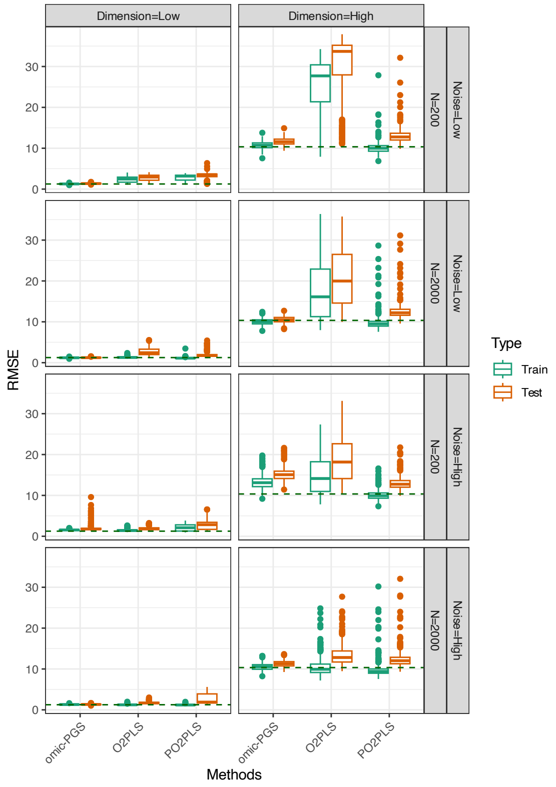

(b) Design III with $r^{++} = 20$

Figure S8: **RMSE of training and test datasets stratified by method and scenario in Design I and III using $r^{++} = 20$.** The reference line represents the true errors.
